# Identification of Potential Transcriptomic Markers in Developing Ankylosing Spondylitis: A Meta-Analysis of Gene Expression Profiles

**DOI:** 10.1155/2015/826316

**Published:** 2015-01-22

**Authors:** Fang Fang, Jian Pan, Lixiao Xu, Gang Li, Jian Wang

**Affiliations:** Institute of Pediatric Research, Children's Hospital of Soochow University, Suzhou 215003, China

## Abstract

The goal of this study was to identify potential transcriptomic markers in developing ankylosing spondylitis by a meta-analysis of multiple public microarray datasets. Using the INMEX (integrative meta-analysis of expression data) program, we performed the meta-analysis to identify consistently differentially expressed (DE) genes in ankylosing spondylitis and further performed functional interpretation (gene ontology analysis and pathway analysis) of the DE genes identified in the meta-analysis. Three microarray datasets (26 cases and 29 controls in total) were collected for meta-analysis. 905 consistently DE genes were identified in ankylosing spondylitis, among which 482 genes were upregulated and 423 genes were downregulated. The upregulated gene with the smallest combined rank product (RP) was *GNG11* (combined RP = 299.64). The downregulated gene with the smallest combined RP was *S100P* (combined RP = 335.94). In the gene ontology (GO) analysis, the most significantly enriched GO term was “immune system process” (*P* = 3.46 × 10^−26^). The most significant pathway identified in the pathway analysis was antigen processing and presentation (*P* = 8.40 × 10^−5^). The consistently DE genes in ankylosing spondylitis and biological pathways associated with those DE genes identified provide valuable information for studying the pathophysiology of ankylosing spondylitis.

## 1. Introduction

Ankylosing spondylitis (AS) represents a chronic inflammatory arthritis, which affects the axial joints such as spine and sacroiliac joints [1]. It causes serious spinal mobility impairment and influences the quality of life [2]. Ankylosing spondylitis is a complex and systemic rheumatic disease; hence systematic screening is required to improve the diagnosis and treatment of ankylosing spondylitis.

Rapid growth of high-throughput transcriptomic data largely enables gene expression profiling and diagnostic targets identification in disease nowadays. In the past decade, several studies have focused on the transcriptional profiling of ankylosing spondylitis using microarrays to identify candidate genes involved in ankylosing spondylitis [3, 4]. Analysis of multiple transcriptomic datasets has the likelihood of discovering robust candidates for diagnosis and treatment. Therefore, we investigated gene expression patterns between ankylosing spondylitis patients and healthy controls in a meta-analysis based on public microarray datasets. The differently expressed genes identified in the meta-analysis were further interpreted by gene ontology analysis and pathway analysis.

To carry out these studies, we used the INMEX (integrative meta-analysis of expression data) program [5]. Careful data procession and annotation were done to insure that the data format and class labels were consistent across datasets. Due to the differences in study design and platform usage, heterogeneity exists among microarray datasets. To address this, we applied the combing rank orders algorithm based on the RankProd package [6], which is robust facing outliers and variations among studies, to carry out the meta-analysis.

## 2. Materials and Methods

### 2.1. Microarray Datasets Search and Selection

In this study, we searched public microarray study till March 18, 2014, according to the keywords “ankylosing spondylitis” in Gene Expression Omnibus (GEO) database (http://www.ncbi.nlm.nih.gov/geo/) [7]. The studies obtained were further selected for the meta-analysis and our selection criteria were (a) case-control study; (b) study providing gene expression data; and (c) study with ankylosing spondylitis patients diagnosed based on the modified New York criteria [8]. Animal studies and studies not about ankylosing spondylitis were excluded in this meta-analysis.

Two investigators independently collected data from each eligible study. The data were composed of GEO accession, sample size, sample source, platform, and gene expression data. Through checking between the two investigators, a final data collection was determined.

### 2.2. Meta-Analysis Methods

According to the data collected from each eligible microarray study, we performed an overall meta-analysis to identify differentially expressed (DE) genes in ankylosing spondylitis. In this study, we used the INMEX (integrative meta-analysis of expression data) program (http://www.inmex.ca/INMEX/) [5] to carry out the meta-analysis.

All eligible datasets were uploaded to INMEX, then processed, and annotated to insure that the data format and class labels were consistent across datasets. After data integrity check, we carried out a meta-analysis using combing rank orders algorithm, with 100 times of permutation tests. The combing rank order algorithm is based on the RankProd package [6] and is robust facing outliers and variations among studies.

### 2.3. Functional Interpretation Methods

Functional interpretation (gene ontology analysis and pathway analysis) of the DE genes identified in the meta-analysis was further performed using the INMEX program. In gene ontology (GO) analysis, a *P* value threshold of 0.05 was used to identify significantly enriched GO terms [9]. In pathway analysis, enrichment analysis was carried out using the hypergeometric test with a *P* value threshold of 0.05 based on the KEGG database [10].

## 3. Results

### 3.1. Studies and Data Included in This Meta-Analysis

**Figure 1:**
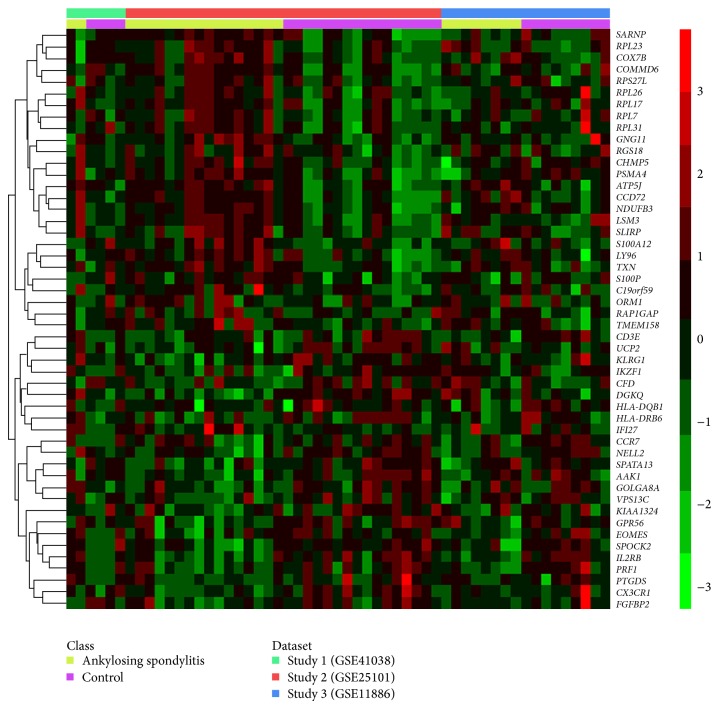
Heat map for visualization of gene pattern changes across different datasets (row-wise comparison). Individual datasets were rescaled in this map to prevent the influence of study-specific effects.

Original search identified 8 studies in total. Then, 5 studies were excluded among which 4 were not about the DE genes between ankylosing spondylitis patients and healthy controls, and 1 was animal study. Through searching and selection, a final list of 3 microarray datasets [3, 4] was collected for meta-analysis. In total, the 3 eligible datasets consisted of 26 cases and 29 controls. All 3 datasets provided case-control data with various sample sources (1 dataset of synovial biopsies sample and 2 datasets of blood sample). The detailed information of these 3 datasets is presented in Table 1. Heat map of rescaled individual expression data for a subset of genes across the 3 datasets is shown in Figure 1, and the patterns of change for a gene among different datasets could be visualized.

### 3.2. Meta-Analysis Results

**Figure 2:**
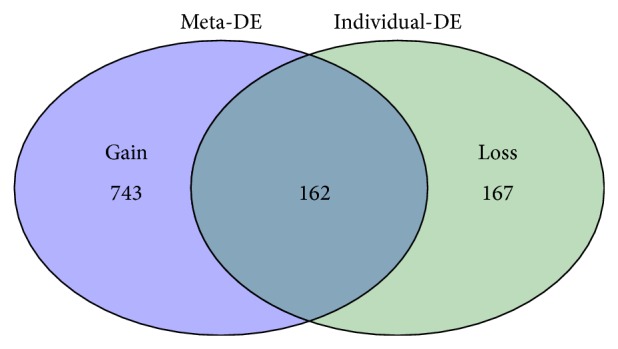
Venn diagram for overlap visualization between meta-analysis results and individual dataset analysis results. Meta-DE: DE genes identified in meta-analysis; individual-DE: DE genes identified in individual dataset analysis.

In this study we performed the meta-analysis based on combing rank orders, and the DE genes with *P* value < 0.05 were selected. Overall, there were 743 gained genes and 167 lost genes in this meta-analysis (Figure 2, see Supplementary Table 1 in Supplementary Materials available online at http://dx.doi.org/10.1155/2014/826316). Gain genes are those identified to be differentially expressed uniquely in the meta-analysis. The expression profiles of gain genes are relatively weak but consistent across datasets. They benefit by larger sample size and hence are more reliable DE genes. Lost genes are those identified to be differentially expressed in individual analysis rather than in the meta-analysis. The expression profiles of lost genes vary largely across different datasets [5].

In total, according to the results of our meta-analysis, 905 genes were identified to be differentially expressed between ankylosing spondylitis patients and healthy controls across microarray datasets (Supplementary Table 2). Among the 905 DE genes, 482 genes were upregulated and 423 genes were downregulated. The top 10 most significantly upregulated genes and top 10 most significantly downregulated genes are shown in Table 2. The upregulated gene with the smallest combined rank product (RP) was* GNG11* (combined RP = 299.64). GNG11, guanine nucleotide binding protein (G protein) gamma 11, is a member of the G protein gamma family which functions in the transmembrane signaling system and cellular senescence [11, 12]. The downregulated gene with the smallest combined RP was* S100P* (combined RP = 335.94). S100P (S100 calcium binding protein P) belongs to the S100 calcium-binding protein family and functions in the regulation of diverse cellular processes [13]. However, neither* GNG11* nor* S100P* have been reported to be associated with ankylosing spondylitis yet.

Many consistently DE genes across datasets identified are involved in immune regulation, such as* COMMD6*,* C19orf59*,* CCR7*,* CX3CR1*,* CFD*, and* FGFBP2* (see Table 2). Although these results are suggestive rather than straightforward, those DE genes could be involved in the pathogenesis of ankylosing spondylitis and further research is required.

### 3.3. Advanced Analyses Results

**Figure 3:**
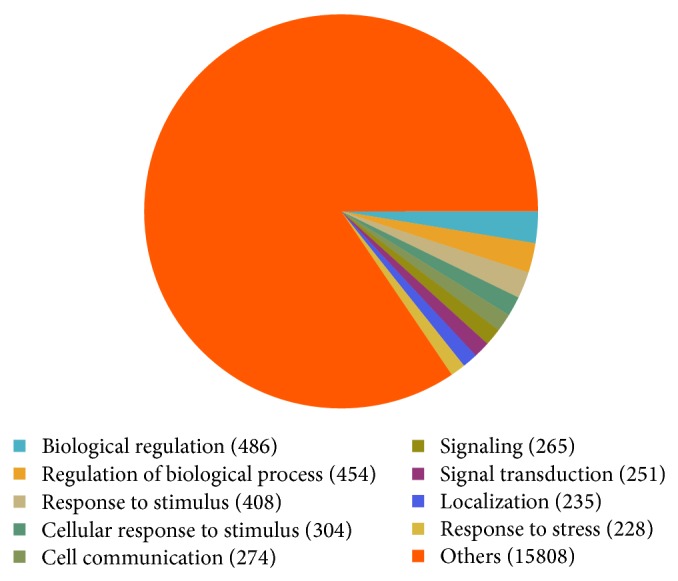
Summary of the GO analysis results for the DE genes in ankylosing spondylitis.

Advanced analyses (GO analysis and pathway analysis) were carried out for further functional investigation of the DE genes.  Figure 3 presented a summary of the GO analysis results. In the GO analysis, 715 GO terms were significantly enriched for the DE genes (see Supplementary Table 3), and the three most significantly enriched GO terms were “immune system process” (*P* = 3.46 × 10^−26^), “immune response” (*P* = 7.83 × 10^−24^), and “defense response” (*P* = 6.99 × 10^−16^) (Table 3). In the pathway analysis, 15 significant pathways were identified when we mapped the DE genes to the KEGG database (see supplementary Table 4), and the three most significant pathways were antigen processing and presentation (*P* = 8.40 × 10^−5^), measles (*P* = 3.30 × 10^−3^), and cell adhesion molecules (CAMs) (*P* = 5.66 × 10^−3^) (Table 4).

## 4. Discussion

A number of genes have been reported to be upregulated or downregulated in ankylosing spondylitis patients [14–16]. Identification of the most important candidate genes and pathways involved in ankylosing spondylitis pathogenesis is a challenge currently. Growing high-throughput transcriptomic data enables meta-analysis of multiple datasets which has the likelihood of discovering robust candidates for diagnosis and treatment. Hence in this study, we performed a meta-analysis of multiple public microarray datasets to identify potential transcriptomic markers in developing ankylosing spondylitis.

In the meta-analysis, 905 consistently DE genes were identified in ankylosing spondylitis, among which 482 genes were upregulated and 423 genes were downregulated. The upregulated gene with the smallest combined RP was* GNG11*. GNG11 belongs to the G protein gamma family and plays a role in the transmembrane signaling system and cellular senescence [11, 12]. The downregulated gene with the smallest combined RP was* S100P*.* S100P* encodes a protein which is a member of the S100 calcium-binding protein family and functions in the regulation of diverse cellular processes [13]. So far,* S100P* is reported to be involved in the development and progression of various cancers [13]. Although the exact contributions of these DE genes to ankylosing spondylitis development are not clear yet, further research is necessary as those genes could be potential transcriptomic markers for ankylosing spondylitis.

Many consistently DE genes identified are involved in immune regulation, suggesting its major role in ankylosing spondylitis development. A set of DE genes without previous studies in ankylosing spondylitis were also identified in our analysis. Some of these DE genes may function in the pathogenesis of ankylosing spondylitis and could be potential biomarkers.

In the GO analysis, 715 significantly enriched GO terms were identified in total. The top three significantly enriched GO terms were “immune system process,” “immune response,” and “defense response.” Similar features were also shown in a previous microarray analysis of ankylosing spondylitis mouse model [17]. The overlap of enriched GO terms between human study and mouse study suggests that immune response plays a major role in ankylosing spondylitis development and therefore deserves further studies. In the pathway analysis, 15 significant pathways were identified in all. The top three significant pathways were antigen processing and presentation, measles, and cell adhesion molecules (CAMs). Our result that pathways involved in immune response are related with the pathogenesis of ankylosing spondylitis is consistent with previous reports highlighting innate immune stimulation and the IL-23 pathway in ankylosing spondylitis pathogenesis [18, 19]. Our analysis also identified pathways not previously studied in ankylosing spondylitis, some of which may harbor interesting functions and deserve further investigation.

In addition, all the results of our meta-analysis should be considered prudently due to the existence of several limitations. One limitation is the insufficient sample size used in our meta-analysis. A second limitation is the lack of subgroup analyses based on potential influential factors, including age, sex, treatment, disease severity, and platform usage, as ankylosing spondylitis is reported to be more prevalent in men and often occur in the third decade of life [1]. The third limitation is that biological knowledge base and pathway information are far from being complete at present and need further investigation. Hence, in order to achieve a more convincible conclusion, further analysis using larger sample size and more complete biological knowledge base and pathway information is required, and stratified analyses on different factors such as age, sex, disease severity, and platform usage are needed. In addition, experimental verification of the candidate DE genes identified should also be performed in the future, and functional studies need to be carried out as well to address the exact roles of those candidate DE genes in ankylosing spondylitis.

In conclusion, we identified consistently DE genes in ankylosing spondylitis that could potentially serve as transcriptomic markers. GO and pathway analyses revealed that those candidates strongly were associated with immune system process besides the underlying complex and multifactor-influenced molecular mechanism. These results provide novel insights into the pathogenesis of ankylosing spondylitis and promote the generation of diagnostic gene sets.

## Supplementary Material

Supplementary Table 1: The 743 gained genes and 167 lost genes in this meta-analysis.
Supplementary Table 2: The 905 genes identified to be differentially expressed between ankylosing spondylitis patients and healthy controls across microarray datasets in this meta-analysis.
Supplementary Table 3: The 715 significantly enriched GO terms for the DE genes in ankylosing spondylitis.
Supplementary Table 4: The 15 significant pathways identified when the DE genes in ankylosing spondylitis were mapped to the KEGG database.

## Figures and Tables

**Table 1:** Studies and data included in this meta-analysis.

Study	GEO accession	Sample size		Sample source	Platform
		AS case	Control		
1	GSE41038	2	4	Synovial biopsies	GPL6883 Illumina HumanRef-8 v3.0 expression beadchip
2	GSE25101	16	16	Blood	GPL6947 Illumina HumanHT-12 V3.0 expression beadchip
3	GSE11886	8	9	Blood	GPL570 Affymetrix Human Genome U133 Plus 2.0 Array

GEO: Gene Expression Omnibus; AS: ankylosing spondylitis.

**Table 2:** The 10 most significantly upregulated and 10 most significantly downregulated genes in ankylosing spondylitis.

Entrez ID	Gene symbol	Gene full name	Combined rank product	Average log fold change
10 most significantly upregulated genes				
2791	*GNG11 *	Guanine nucleotide binding protein (G protein), gamma 11	299.64	0.380467
51372	*TMA7 *	Translation machinery associated 7 homolog (*S. cerevisiae*)	312.39	0.610581
9349	*RPL23 *	Ribosomal protein L23	324.13	0.148532
23643	*LY96 *	Lymphocyte antigen 96	330.33	0.195432
170622	*COMMD6 *	COMM domain containing 6	332.47	0.293187
5909	*RAP1GAP *	RAP1 GTPase activating protein	343.66	0.223733
1349	*COX7B *	Cytochrome c oxidase subunit VIIb	371.12	0.132367
6283	*S100A12 *	S100 calcium binding protein A12	439.12	0.334567
27258	*LSM3 *	LSM3 homolog, U6 small nuclear RNA associated (*S. cerevisiae*)	440.15	0.596028
199675	*C19orf59 *	Chromosome 19 open reading frame 59	494.30	0.358146

10 most significantly downregulated genes				
6286	*S100P *	S100 calcium binding protein P	335.94	−0.199948
3560	*IL2RB *	Interleukin 2 receptor, beta	351.04	−0.166370
221178	*SPATA13 *	Spermatogenesis associated 13	473.13	−0.442046
9289	*GPR56 *	G protein-coupled receptor 56	528.50	−0.239562
22848	*AAK1 *	AP2 associated kinase 1	530.65	−0.364595
5551	*PRF1 *	Perforin 1 (pore forming protein)	531.02	−0.114758
1236	*CCR7 *	Chemokine (C-C motif) receptor 7	609.87	−0.199449
1524	*CX3CR1 *	Chemokine (C-X3-C motif) receptor 1	622.12	−0.351950
1675	*CFD *	Complement factor D (adipsin)	636.15	−0.365854
83888	*FGFBP2 *	Fibroblast growth factor binding protein 2	653.39	−1.054696

**Table 3:** The 10 most significantly enriched GO terms for the DE genes in ankylosing spondylitis.

ID	Term	*P* value
GO:0002376	Immune system process	3.46*E* − 26
GO:0006955	Immune response	7.83*E* − 24
GO:0006952	Defense response	6.99*E* − 16
GO:0002682	Regulation of immune system process	3.01*E* − 13
GO:0006950	Response to stress	2.02*E* − 12
GO:0050776	Regulation of immune response	1.44*E* − 11
GO:0001775	Cell activation	2.24*E* − 11
GO:0045087	Innate immune response	5.19*E* − 11
GO:0045321	Leukocyte activation	1.32*E* − 10
GO:0030097	Hemopoiesis	2.91*E* − 10

**Table 4:** The 10 most significant pathways identified when the DE genes were mapped to the KEGG database.

Pathway	*P* value
Antigen processing and presentation	0.0000840
Measles	0.0032959
Cell adhesion molecules (CAMs)	0.0056626
Jak-STAT signaling pathway	0.0070316
Hypertrophic cardiomyopathy (HCM)	0.0099861
Pathogenic *Escherichia coli* infection	0.0105380
T cell receptor signaling pathway	0.0167490
Natural killer cell mediated cytotoxicity	0.0172540
Graft-versus-host disease	0.0174410
Allograft rejection	0.0186970
